# Chronic recurrent multifocal osteomyelitis mimicking migraine

**DOI:** 10.1136/pn-2022-003522

**Published:** 2022-09-07

**Authors:** Akira Taniguchi, Yasutaka Ichikawa, Masayuki Maeda, Hidekazu Tomimoto

**Affiliations:** 1 Neurology, Graduate School of Medicine Faculty of Medicine, Mie University, Tsu, Japan; 2 Department of Radiology, Graduate School of Medicine Faculty of Medicine, Mie University, Tsu, Mie, Japan; 3 Department of Neuroradiology, Graduate School of Medicine Faculty of Medicine, Mie University, Tsu, Mie, Japan

**Keywords:** MIGRAINE

A 22-year-old woman developed left-sided orbitotemporal headache with swelling at the temple and lacrimation around the left eye. A few years before, she had a 2-month history of daily left-sided headaches with photosensitivity, but with no aura or associated nausea and vomiting. She had consulted a headache specialist and took a triptan for migraine but without benefit. One year before presentation, she had developed a similar pain with ptosis. She had no family history of migraines. An MR scan of the brain was normal, and her symptoms resolved spontaneously. On further questioning, she reported sternum pain for a few months.

On examination, there was mild swelling and tenderness in the left orbitotemporal region. Investigations included mildly elevated serum C reactive protein (8 mg/L) and erythrocyte sedimentation rate (49 mm/hour).

A CT scan of the head identified osteolytic and osteosclerotic changes in the sphenoidal, frontal and parietal bones on the left side (figure 1A). An MR scan of the brain showed T2/fluid-attenuated inversion recovery (FLAIR) hyperintensities in the sphenoidal bone, and the temporal and pterygoid muscles. A gadolinium-enhanced MR scan of the brain showed thickening and enhancement of the dura mater in the left frontal region (figure 1B, lower row). A retrospective review of previous MR identified T2/FLAIR hyperintensities in the sphenoidal bone and temporal muscle (figure 1B, upper row). Bone scintigraphy showed increased uptake in both clavicles, the sternum and sphenoidal and frontal bones on the left side (figure 1C). Based on her history and imaging findings, we diagnosed chronic recurrent multifocal osteomyelitis (CRMO).

**Figure 1:**
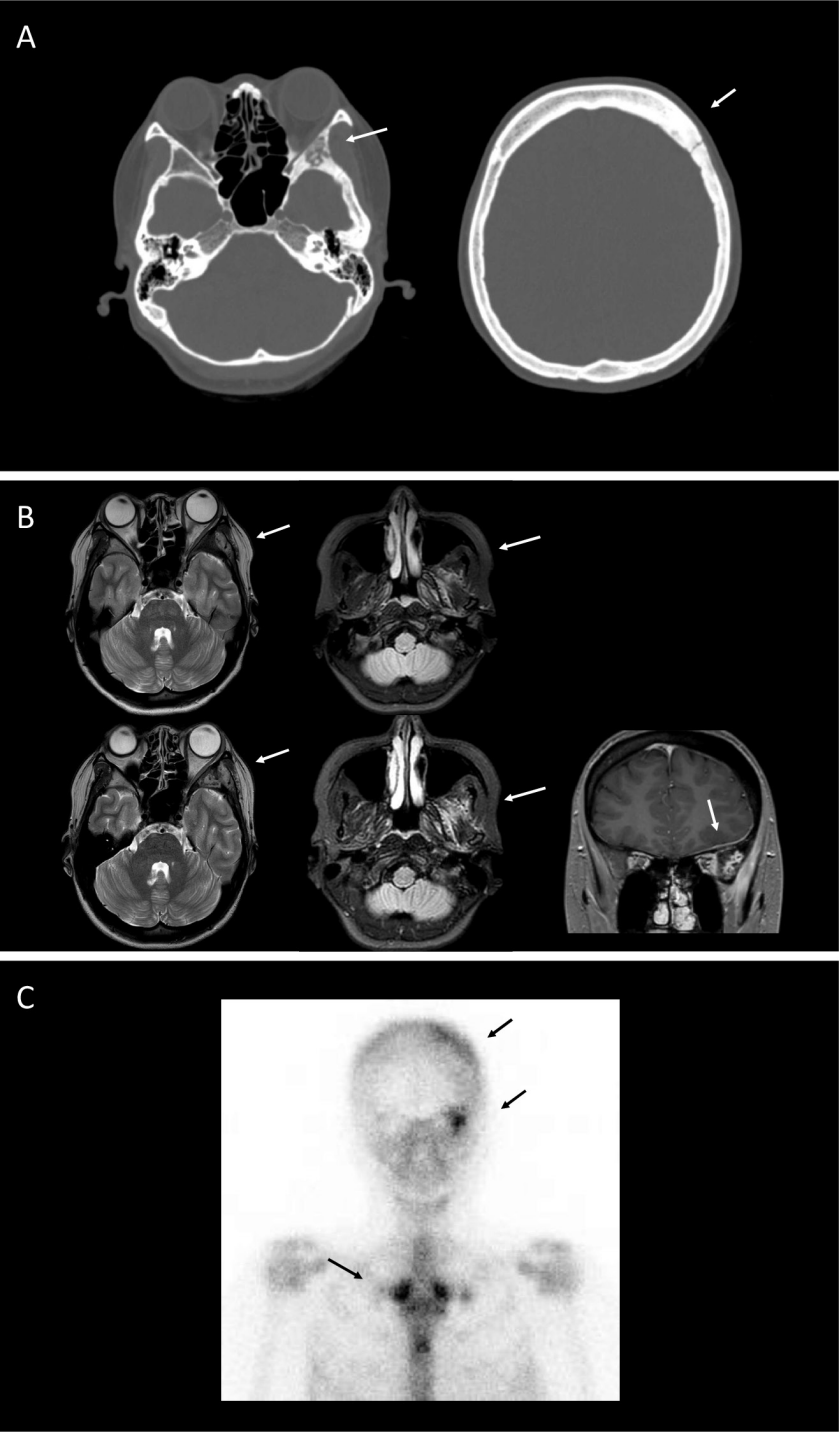
(A) A CT scan of the skull showing osteolytic and osteosclerotic changes in the sphenoidal, frontal and parietal bones on the left side (arrows). (B) An MR scan of the brain: initial (upper row) and current (lower row) presentation. Images show T2 hyperintensity in the sphenoidal bone (upper and lower left, arrow), fluid-attenuated inversion recovery hyperintensity in the temporal and pterygoid muscles (upper right and lower middle, arrow), and thickening and gadolinium enhancement of the dura mater in the left frontal region (lower right, arrow). (C) Bone scintigraphy showing increased uptake in both clavicles, sternum, and sphenoidal and frontal bones on the left side (arrows).

CRMO is an autoimmune disorder that primarily affects children and adolescents, and with a female predominance. It is characterised by a self-limited course, with recurrences of non-infective skeletal inflammations. SAPHO syndrome, a combination of synovitis, acne, pustulosis, hyperostosis and osteitis, is considered the same disease process. Although CRMO is easily recognised if these symptoms are associated with bone pain, their development time is variable.^1^ This patient had no associated symptoms on presentation, but further questions identified chest pain that had developed a few years before.

The common sites of CRMO include the long bone metaphases, but there are other reported cases involving the frontal and temporal areas of the skull.^2–4^ In this patient, pain around the left orbitotemporal region was the likely reason for the initial suspicion of migraine attacks. In addition to headaches, CRMO may have various neurological complications, including aseptic meningitis, and hypertrophic pachymeningitis.^3 5^ This patient developed unilateral ptosis in the previous year and lacrimation on presentation, which are also common in migraines.^6^ There were no other trigeminal autonomic symptoms, such as conjunctival injection. Given the probable diagnosis of CRMO, ptosis and lacrimation were most likely caused by the spread of local inflammation around the skin and lacrimal gland. Since there was no aura, accompanying symptoms, family history or benefit from triptans, we felt able to rule out migraines.

CRMO may present with headaches, in addition to other neurological symptoms. Neurologists should keep this in mind when considering possible differentials of headache in young people.

**Key points**

- Chronic recurrent multifocal osteomyelitis (CRMO) is a rare autoimmune skeletal disorder, primarily affecting children and adolescents, with a female predominance.
- Patients with CRMO may also be misdiagnosed with migraine if they have temple pain and autonomic symptoms such as ptosis and lacrimation.
- Neurologists should consider this condition when managing headache in young people.

## Data Availability

All data relevant to the study are included in the article or uploaded as supplemental information.
